# Finished Genome Sequence of *Escherichia coli* K-12 Strain HMS174 (ATCC 47011)

**DOI:** 10.1128/genomeA.00975-14

**Published:** 2014-11-20

**Authors:** Juergen Mairhofer, Peter M. Krempl, Gerhard G. Thallinger, Gerald Striedner

**Affiliations:** a Bioinformatics Group, Institute for Knowledge Discovery, Graz University of Technology, Graz, Austria; b Department of Biotechnology, University of Natural Resources and Life Sciences, Vienna, Austria; c Austrian Centre of Industrial Biotechnology (ACIB GmbH), Graz, Austria

## Abstract

*Escherichia coli* strain K-12 substrain HMS174 is an engineered descendant of the *E. coli* K-12 wild-type strain. Like its ancestor, it is an important organism in biotechnological research and is used in fermentation processes for heterologous protein production. Here, we report the complete genome sequence of *E. coli* HMS174 (ATCC 47011).

## GENOME ANNOUNCEMENT

The prototrophic *Escherichia coli* HMS174 was derived by mating a *thy-1* version of *E. coli* strain K-12 substrain W3110 with *Escherichia coli* strain K-12 substrain KL16-99 Hfr by Campbell et al. (1, 2), and it carries the following modifications: *F*^−^, *λ*^−^, *recA1*, *rpoB331*, and *hsdR19*. HMS174 was originally used for the refinement of the structural and genetic map of the T7 phage (1) in the Studier lab at Brookhaven and later on became a popular strain for the expression of recombinant proteins after lysogenization with the DE3 prophage. *E. coli* HMS174 provides a *recA1* mutation in a K-12 background. Due to this mutation, certain target genes whose products may cause the loss of the DE3 prophage become stabilized. However, the actual genome sequence remains to be elucidated.

The genome of *E. coli* strain K-12 substrain HMS174 was sequenced using a combination of next-generation sequencing methods. A first draft assembly based on the sequences of an 8-kbp paired-end library (Roche 454 GS FLX Titanium; 718,718 reads, for a total of 108.8 Mb; 23-fold coverage; ZMF, Medical University of Graz, Austria) generated with Newbler 2.6 consisted of 165 contigs, 81 of which were joined into a single circular scaffold. The gaps resulting from repetitive sequences were resolved by *in silico* gap filling. The remaining gaps were closed by PCR, followed by Sanger sequencing, yielding a draft genome of 4,583,580 bp. To improve the quality of the sequence by eliminating the 454 sequencing errors in the homopolymer stretches, the genome was subsequently sequenced using the Illumina paired-end method (Illumina HiSeq 2000; 16,836,098 reads, for a total of 1.68 Gb; 367-fold coverage; Ambry Genetics, Aliso Viejo, CA). The Illumina reads were aligned to the draft genome with CLC Genomics Workbench 4.7.1 (CLC bio, Aarhus, Denmark). Conflicts in the consensus sequence were resolved by voting.

The genomic structure of HMS174 was compared with that of the reference *E. coli* K-12 strains MG1655 and W3110 using Mauve (3), showing that HMS174 does not contain genomic regions without homologs in one of those strains. Functional annotation was performed by matching the genes of these reference strains based on sequence similarity, using EcoCyc (4) as the primary data source. Additionally, the origin of replication was predicted with OriginX (5). Clustered regularly interspaced short palindromic repeats (CRISPRs) were predicted with CRT (6).

The final genome includes 4,584,860 bases, with a G+C content of 50.82%. The annotation totals 4,437 putative genes, 4,241 of which are protein coding. There are seven instances of the ribosomal 5S-23S-16S cluster, an additional 5S rRNA gene, 85 tRNAs, one transfer-messenger RNA (tmRNA), and 88 RNA genes of regulatory or miscellaneous function. Two CRISPRs were detected with 12 and 6 spacers, respectively.

The closest sequenced K-12 strain is *E. coli* W3110 (7). In relation to W3110, the HMS174 genome shows 13 deletions (1,198 to 20,110 bp) and 7 insertions (543 to 6,790 bp). Specifically, 9 IS elements and the *ymf*, *abg*, *yda*, *ynf*, and *rbs* operons are deleted. In addition, HMS174 carries the CPZ-55 prophage, 5 additional IS elements (1 IS*3*, 1 IS*4*, 2 IS*5*, and 1 IS*150*), and a lyase containing HEAT-repeat.

### Nucleotide sequence accession number.

The genome sequence for *E. coli* strain K-12 substrain HMS174 has been deposited at the European Nucleotide Archive under the accession no. LM993812.
